# Supplementary material for: Cellular energy regulates mRNA degradation in a codon-specific manner
Source: Mol Syst Biol. 2024 Mar 15;20(5):506–20. doi: 10.1038/s44320-024-00026-9 (PMC11066088; doi:10.1038/s44320-024-00026-9)
Supplement: Supplementary file 1 — Appendix [file 44320_2024_26_MOESM1_ESM.pdf]

Table of Contents

Appendix Figure S1: Relative mRNA half-lives across samples and tissues. 2

Appendix Figure S2: Frequency of the optimal codon CUG in the coding sequence relates to changes in mRNA half-life differently depending on the tissue. 3

Appendix Figure S3: Codons change in their associations with relative mRNA half-life according to their optimality and decoding rate. 5

Appendix Figure S4: COMD coefficient is qualitatively robust to the choice of reference decoding rate used to compute it. 6

Appendix Figure S5: Mitochondrial-related pathways are enriched for differences in COMD coefficient across and within tissues in human and across cell types in mouse. 7

Appendix Figure S6: The COMD coefficient associates with ischemic time for individuals of similar age in Lung. 8

Appendix Figure S7: The COMD coefficient associates with ischemic time for individuals of similar age in Heart - Left Ventricle. 9

Appendix Figure S8: The COMD coefficient associates with ischemic time for individuals of similar age in Adrenal Gland. 10

Appendix Figure S9: The COMD coefficient associates with ischemic time for individuals of similar age in Skin - Not Sun Exposed (Suprapubic). 11

Appendix Figure S10: The COMD coefficient associates with ischemic time for individuals of similar age in Muscle - Skeletal. 12

Appendix Figure S11: Codon-associated 5' coverage correlates with codon optimality and decoding rate metrics. 13

Appendix Figure S12: Ternary complex concentration increases asymptotically with ATP concentration. 14

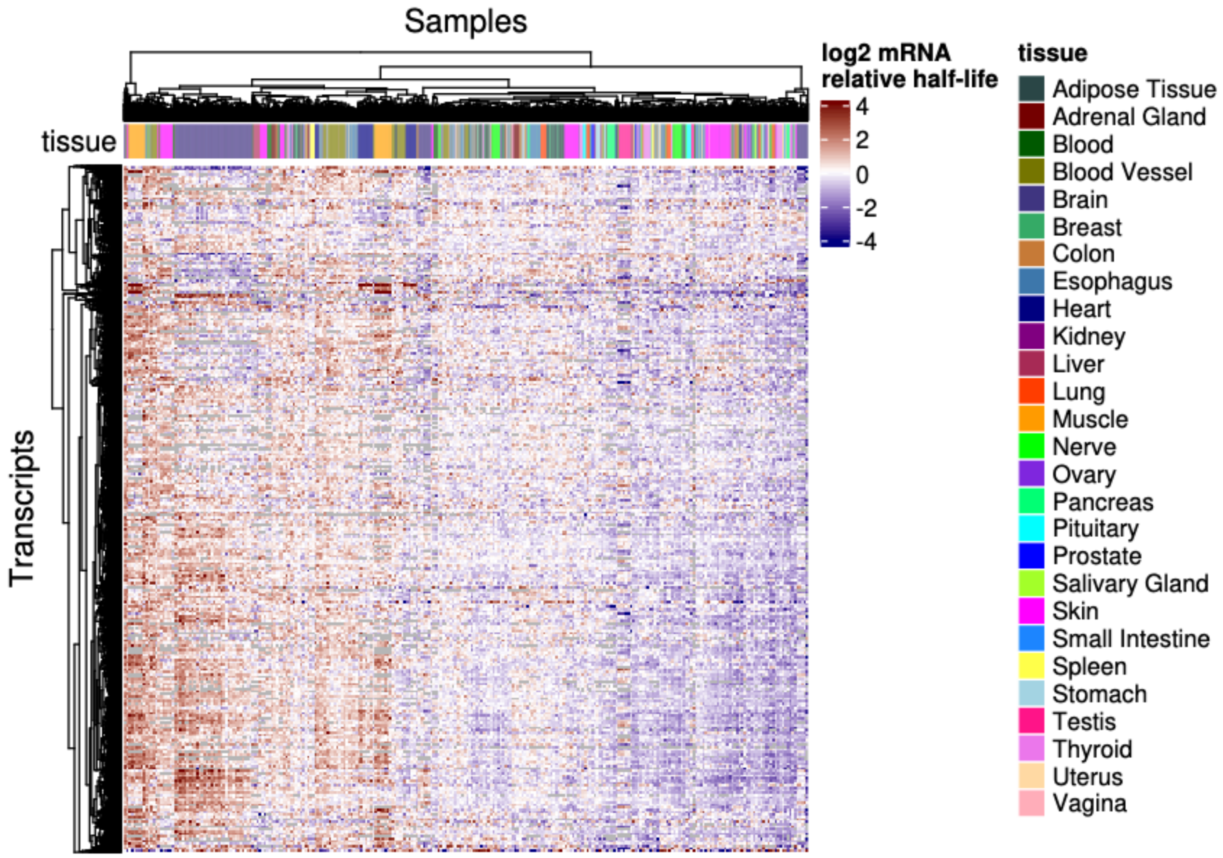

Appendix Figure S1: Relative mRNA half-lives across samples and tissues.
The columns represent transcript major isoforms expressed in at least ⅔ of the samples. $\log_2$ relative mRNA half-life was saturated at -4 and 4. Each sample is colored by the major tissue it belongs to.

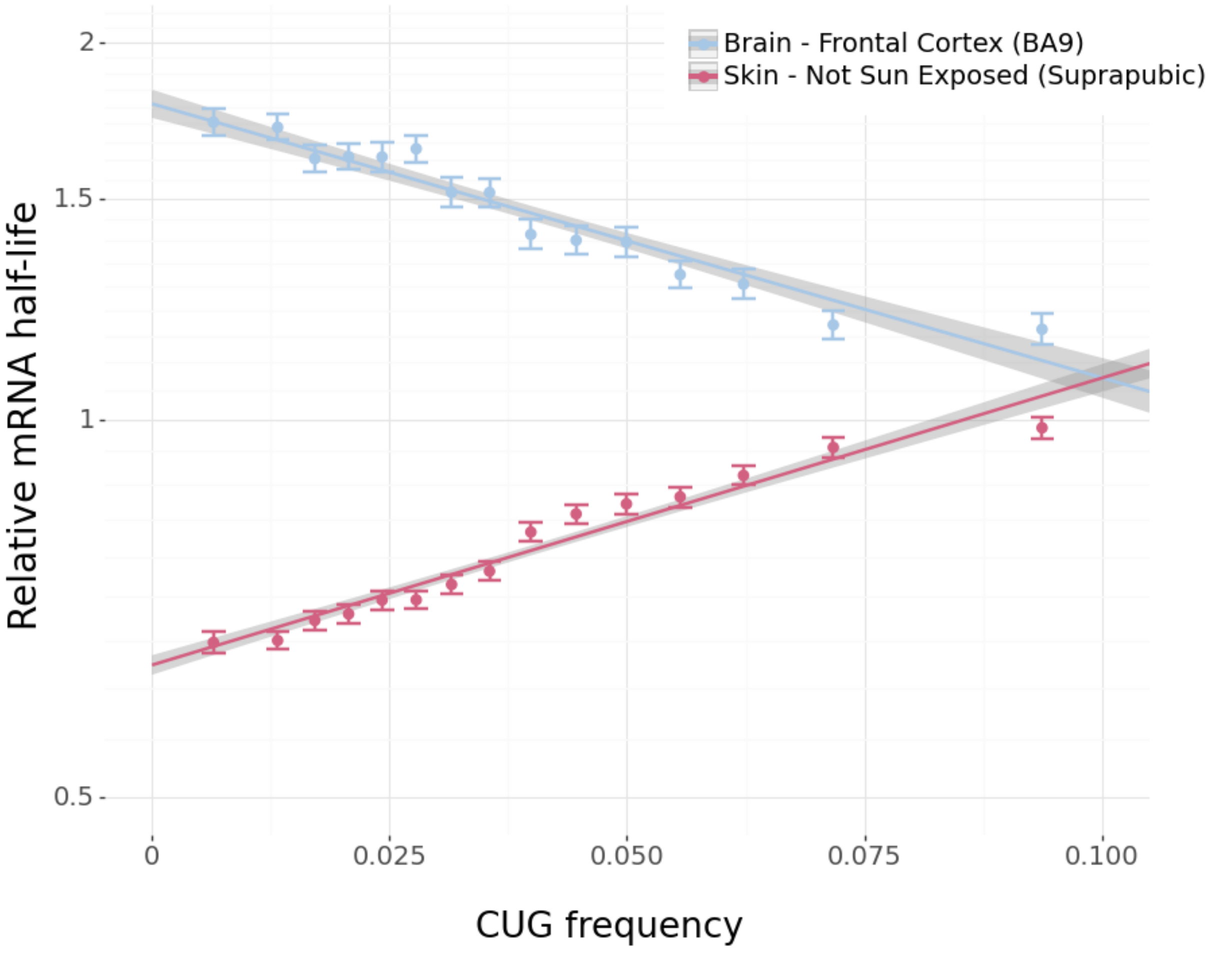

Appendix Figure S2: Frequency of the optimal codon CUG in the coding sequence relates to changes in mRNA half-life differently depending on the tissue.

The mean and standard error bars of relative mRNA half-life are plotted for each group of mRNAs within equally-sized bins of CUG frequency. Transcripts with low usage of CUG exhibit large half-life fold changes between Brain - Frontal Cortex (BA9) and Skin Not Sun-Exposed. In contrast, transcripts with high usage of CUG exhibit a mild half-life fold change. The result of a linear regression of relative mRNA half-life on the frequency of CUG is plotted for each tissue. The slope of each line corresponds to the estimated codon effect of CUG for these tissues reported in the heatmap of Figure 1B.

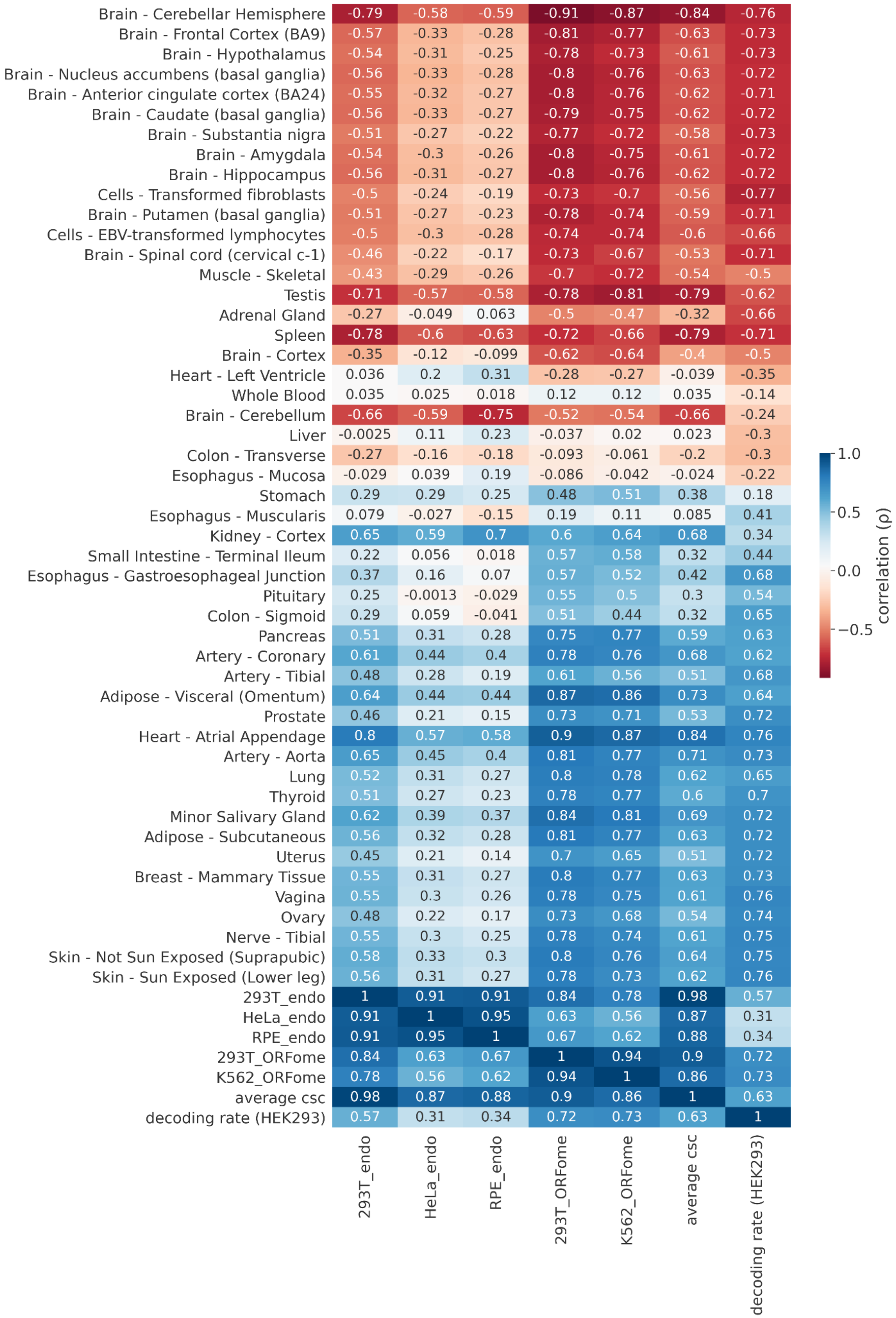
Brain - Cerebellar Hemisphere -0.79 -0.58 -0.59 -0.91 -0.87 -0.84 -0.76
Brain - Frontal Cortex (BA9) -0.57 -0.33 -0.28 -0.81 -0.77 -0.63 -0.73
Brain - Hypothalamus -0.54 -0.31 -0.25 -0.78 -0.73 -0.61 -0.73
Brain - Nucleus accumbens (basal ganglia) -0.56 -0.33 -0.28 -0.8 -0.76 -0.63 -0.72
Brain - Anterior cingulate cortex (BA24) -0.55 -0.32 -0.27 -0.8 -0.76 -0.62 -0.71
Brain - Caudate (basal ganglia) -0.56 -0.33 -0.27 -0.79 -0.75 -0.62 -0.72
Brain - Substantia nigra -0.51 -0.27 -0.22 -0.77 -0.72 -0.58 -0.73
Brain - Amygdala -0.54 -0.3 -0.26 -0.8 -0.75 -0.61 -0.72
Brain - Hippocampus -0.56 -0.31 -0.27 -0.8 -0.76 -0.62 -0.72
Cells - Transformed fibroblasts -0.5 -0.24 -0.19 -0.73 -0.7 -0.56 -0.77
Brain - Putamen (basal ganglia) -0.51 -0.27 -0.23 -0.78 -0.74 -0.59 -0.71
Cells - EBV-transformed lymphocytes -0.5 -0.3 -0.28 -0.74 -0.74 -0.6 -0.66
Brain - Spinal cord (cervical c-1) -0.46 -0.22 -0.17 -0.73 -0.67 -0.53 -0.71
Muscle - Skeletal -0.43 -0.29 -0.26 -0.7 -0.72 -0.54 -0.5
Testis -0.71 -0.57 -0.58 -0.78 -0.81 -0.79 -0.62
Adrenal Gland -0.27 -0.049 0.063 -0.5 -0.47 -0.32 -0.66
Spleen -0.78 -0.6 -0.63 -0.72 -0.66 -0.79 -0.71
Brain - Cortex -0.35 -0.12 -0.099 -0.62 -0.64 -0.4 -0.5
Heart - Left Ventricle 0.036 0.2 0.31 -0.28 -0.27 -0.039 -0.35
Whole Blood 0.035 0.025 0.018 0.12 0.12 0.035 -0.14
Brain - Cerebellum -0.66 -0.59 -0.75 -0.52 -0.54 -0.66 -0.24
Liver -0.0025 0.11 0.23 -0.037 0.02 0.023 -0.3
Colon - Transverse -0.27 -0.16 -0.18 -0.093 -0.061 -0.2 -0.3
Esophagus - Mucosa -0.029 0.039 0.19 -0.086 -0.042 -0.024 -0.22
Stomach 0.29 0.29 0.25 0.48 0.51 0.38 0.18
Esophagus - Muscularis 0.079 -0.027 -0.15 0.19 0.11 0.085 0.41
Kidney - Cortex 0.65 0.59 0.7 0.6 0.64 0.68 0.34
Small Intestine - Terminal Ileum 0.22 0.056 0.018 0.57 0.58 0.32 0.44
Esophagus - Gastroesophageal Junction 0.37 0.16 0.07 0.57 0.52 0.42 0.68
Pituitary 0.25 -0.0013 -0.029 0.55 0.5 0.3 0.54
Colon - Sigmoid 0.29 0.059 -0.041 0.51 0.44 0.32 0.65
Pancreas 0.51 0.31 0.28 0.75 0.77 0.59 0.63
Artery - Coronary 0.61 0.44 0.4 0.78 0.76 0.68 0.62
Artery - Tibial 0.48 0.28 0.19 0.61 0.56 0.51 0.68
Adipose - Visceral (Omentum) 0.64 0.44 0.44 0.87 0.86 0.73 0.64
Prostate 0.46 0.21 0.15 0.73 0.71 0.53 0.72
Heart - Atrial Appendage 0.8 0.57 0.58 0.9 0.87 0.84 0.76
Artery - Aorta 0.65 0.45 0.4 0.81 0.77 0.71 0.73
Lung 0.52 0.31 0.27 0.8 0.78 0.62 0.65
Thyroid 0.51 0.27 0.23 0.78 0.77 0.6 0.7
Minor Salivary Gland 0.62 0.39 0.37 0.84 0.81 0.69 0.72
Adipose - Subcutaneous 0.56 0.32 0.28 0.81 0.77 0.63 0.72
Uterus 0.45 0.21 0.14 0.7 0.65 0.51 0.72
Breast - Mammary Tissue 0.55 0.31 0.27 0.8 0.77 0.63 0.73
Vagina 0.55 0.3 0.26 0.78 0.75 0.61 0.76
Ovary 0.48 0.22 0.17 0.73 0.68 0.54 0.74
Nerve - Tibial 0.55 0.3 0.25 0.78 0.74 0.61 0.75
Skin - Not Sun Exposed (Suprapubic) 0.58 0.33 0.3 0.8 0.76 0.64 0.75
Skin - Sun Exposed (Lower leg) 0.56 0.31 0.27 0.78 0.73 0.62 0.76
293T_endo 1 0.91 0.91 0.84 0.78 0.98 0.57
HeLa_endo 0.91 1 0.95 0.63 0.56 0.87 0.31
RPE_endo 0.91 0.95 1 0.67 0.62 0.88 0.34
293T_ORFome 0.84 0.63 0.67 1 0.94 0.9 0.72
K562_ORFome 0.78 0.56 0.62 0.94 1 0.86 0.73
average csc 0.98 0.87 0.88 0.9 0.86 1 0.63
decoding rate (HEK293) 0.57 0.31 0.34 0.72 0.73 0.63 1
293T_endo
HeLa_endo
RPE_endo
293T_ORFome
K562_ORFome
average csc
decoding rate (HEK293)
1.0
0.5
0.0
−0.5
correlation (ρ)

Appendix Figure S3: Codons change in their associations with relative mRNA half-life according to their optimality and decoding rate.

Each cell of the heatmap represents the correlation between the estimated codon effects for each tissue (rows) and different metrics of codon optimality and decoding rate computed in different cell lines (columns). The labels 293T_endo, Hela_endo, RPE_endo, 293T_ORFome, K562_ORFome correspond to the CSC (codon stability coefficient) in the different cell lines (Hela, 293T, RPE, K562) computed either from endogenous mRNAs (endo) or constructs (ORFome) and were obtained from (Wu et al, 2019). The decoding rate in HEK293 was obtained from (Dana & Tuller, 2015).

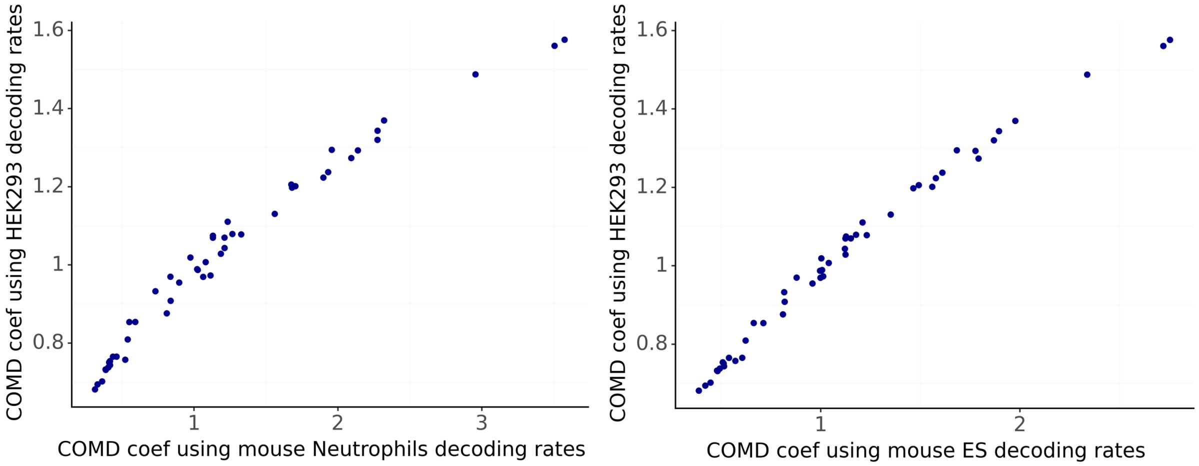

Appendix Figure S4: COMD coefficient is qualitatively robust to the choice of reference decoding rate used to compute it.

COMD coefficient per tissue in GTEx computed using mouse neutrophils and mouse embryonic stem cells codon decoding rates as reference. For each one of the cell lines the ranking of the COMD coefficient is similar to the one computed from HEK293 despite coming from different species, cell lines, Ribo-Seq datasets and laboratories.

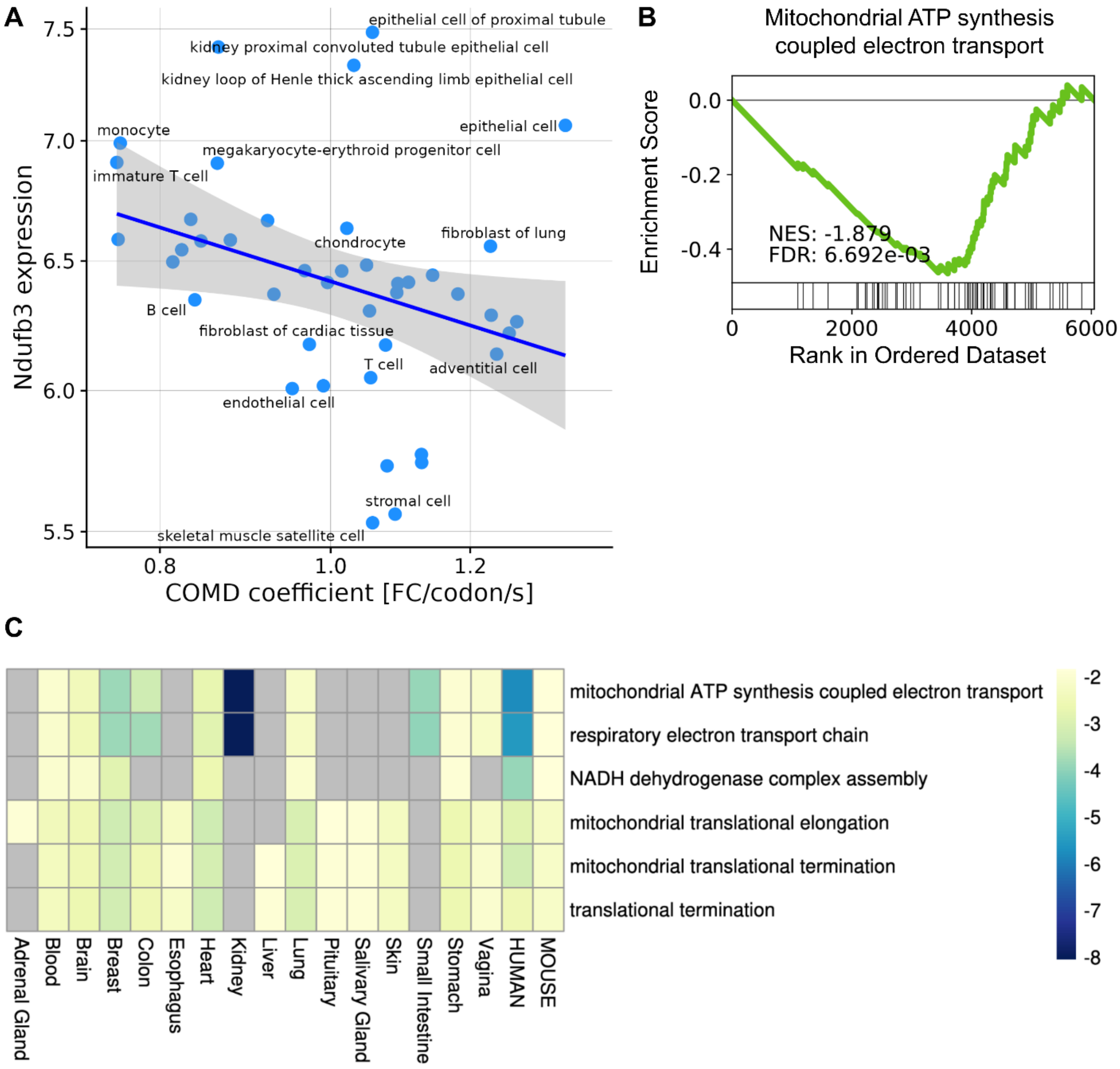

Appendix Figure S5: Mitochondrial-related pathways are enriched for differences in COMD coefficient across and within tissues in human and across cell types in mouse.

A. Relationship between the COMD coefficient and the expression of *Ndufb3*, a gene that encodes a subunit of mitochondrial respiratory complex I, across cell types in mouse (Spearman's rho = -0.44, $P = 0.0034$).

B. Gene set enrichment analysis for the biological process "mitochondrial ATP synthesis coupled electron transport" in mouse.

C. GSEA normalized enrichment score for significant pathways of genes correlated with variations in the COMD coefficient across samples from the same tissue, between tissues in human and between tissues in mouse. Pathways shown are restricted to the ones commonly found in human and mouse.

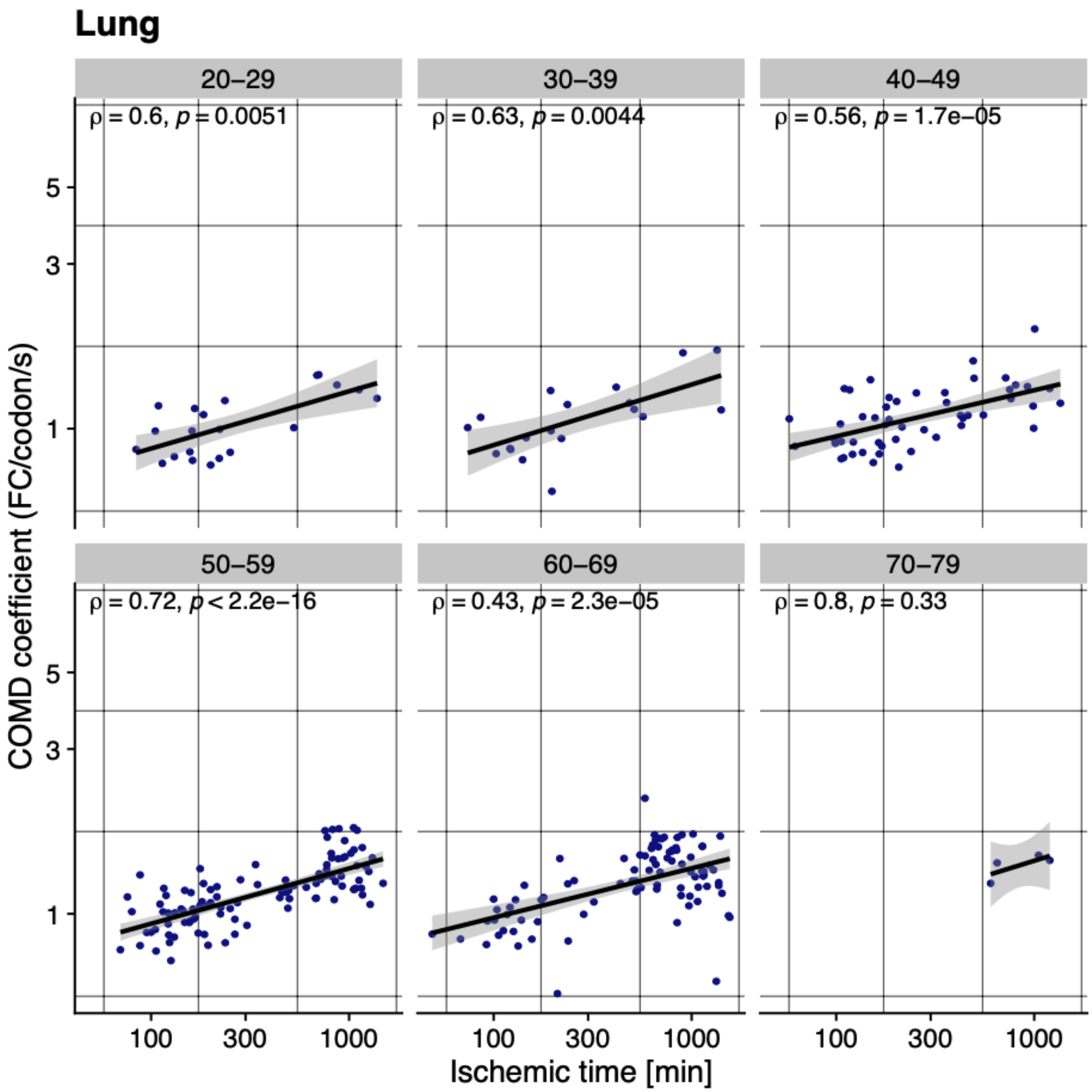

Appendix Figure S6: The COMD coefficient associates with ischemic time for individuals of similar age in Lung.

COMD coefficient against ischemic time (min) for Lung in different age groups.

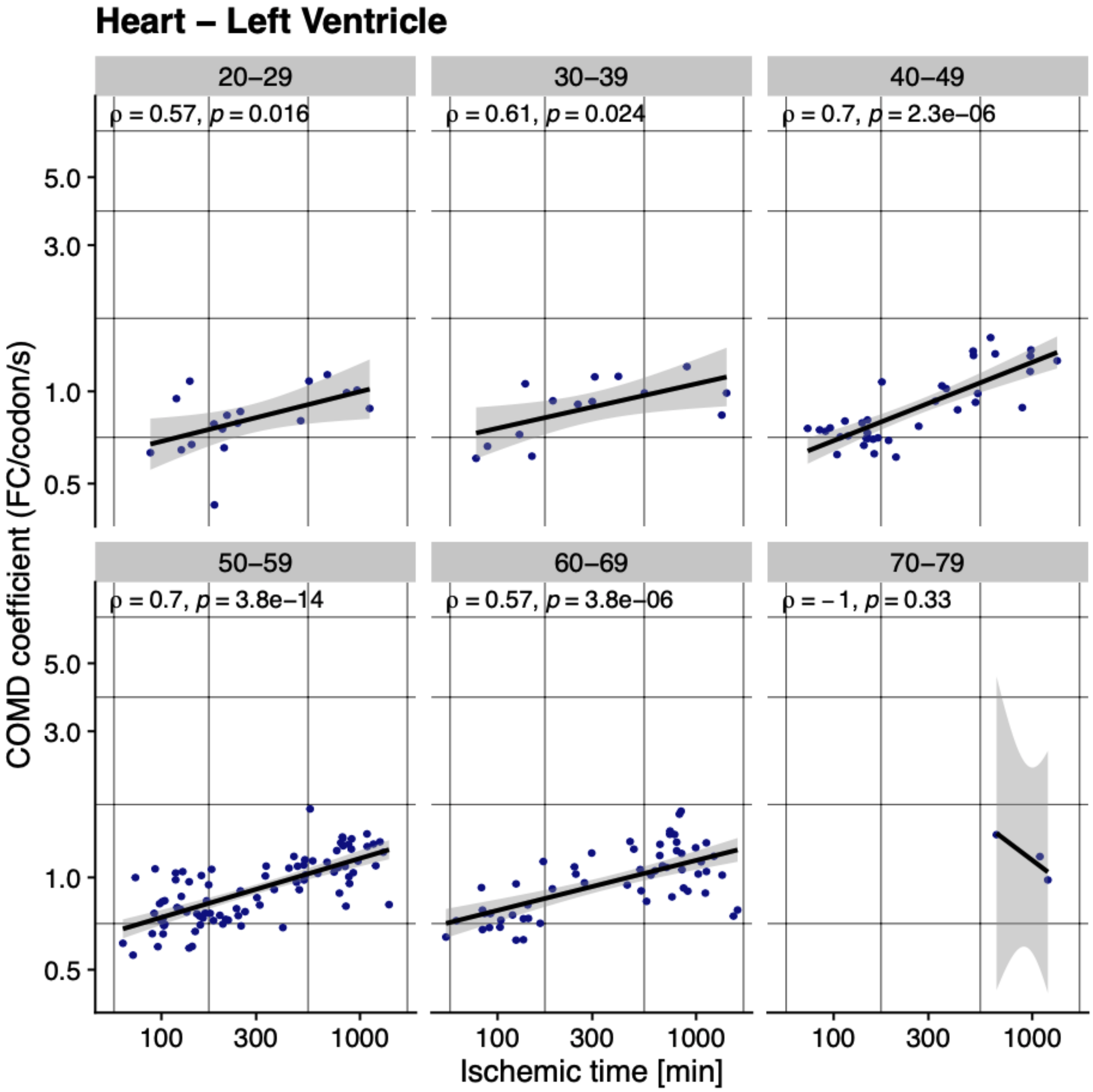

Appendix Figure S7: The COMD coefficient associates with ischemic time for individuals of similar age in Heart - Left Ventricle.

COMD coefficient against ischemic time (min) for Heart - Left Ventricle in different age groups.

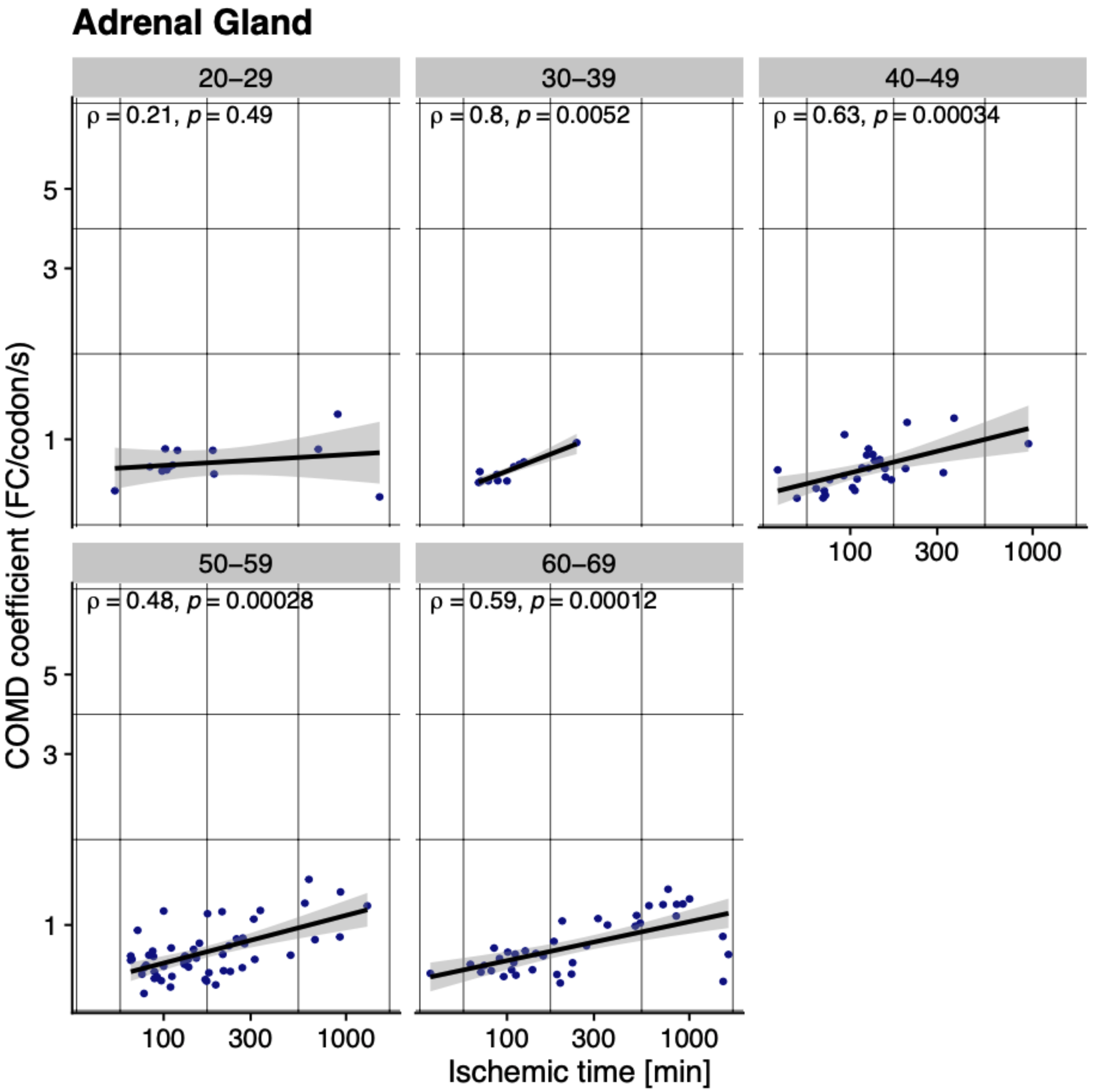

Appendix Figure S8: The COMD coefficient associates with ischemic time for individuals of similar age in Adrenal Gland.

COMD coefficient against ischemic time (min) for Adrenal Gland in different age groups.

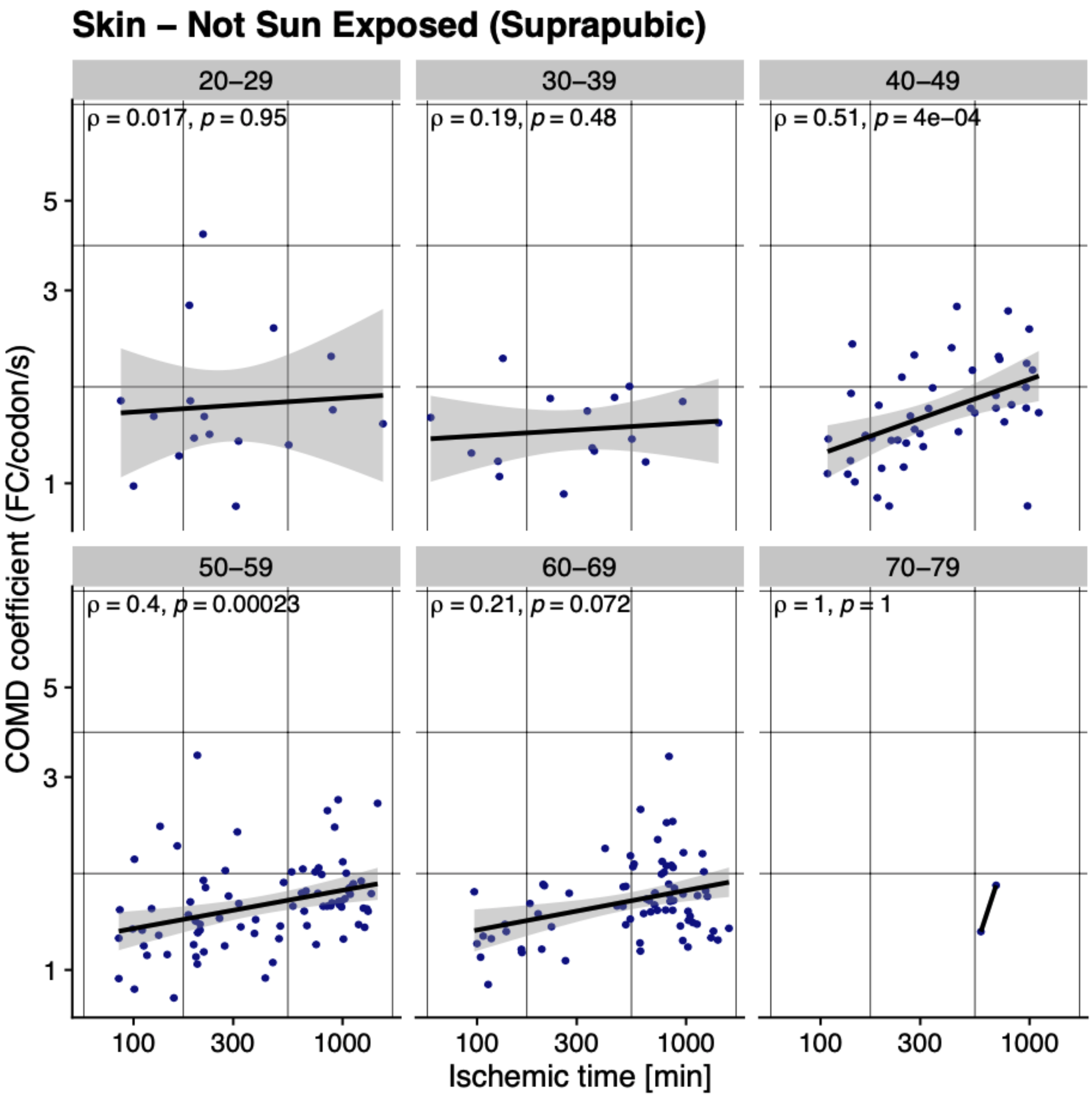

Appendix Figure S9: The COMD coefficient associates with ischemic time for individuals of similar age in Skin - Not Sun Exposed (Suprapubic).

COMD coefficient against ischemic time (min) for Skin - Not Sun Exposed (Suprapubic) in different age groups.

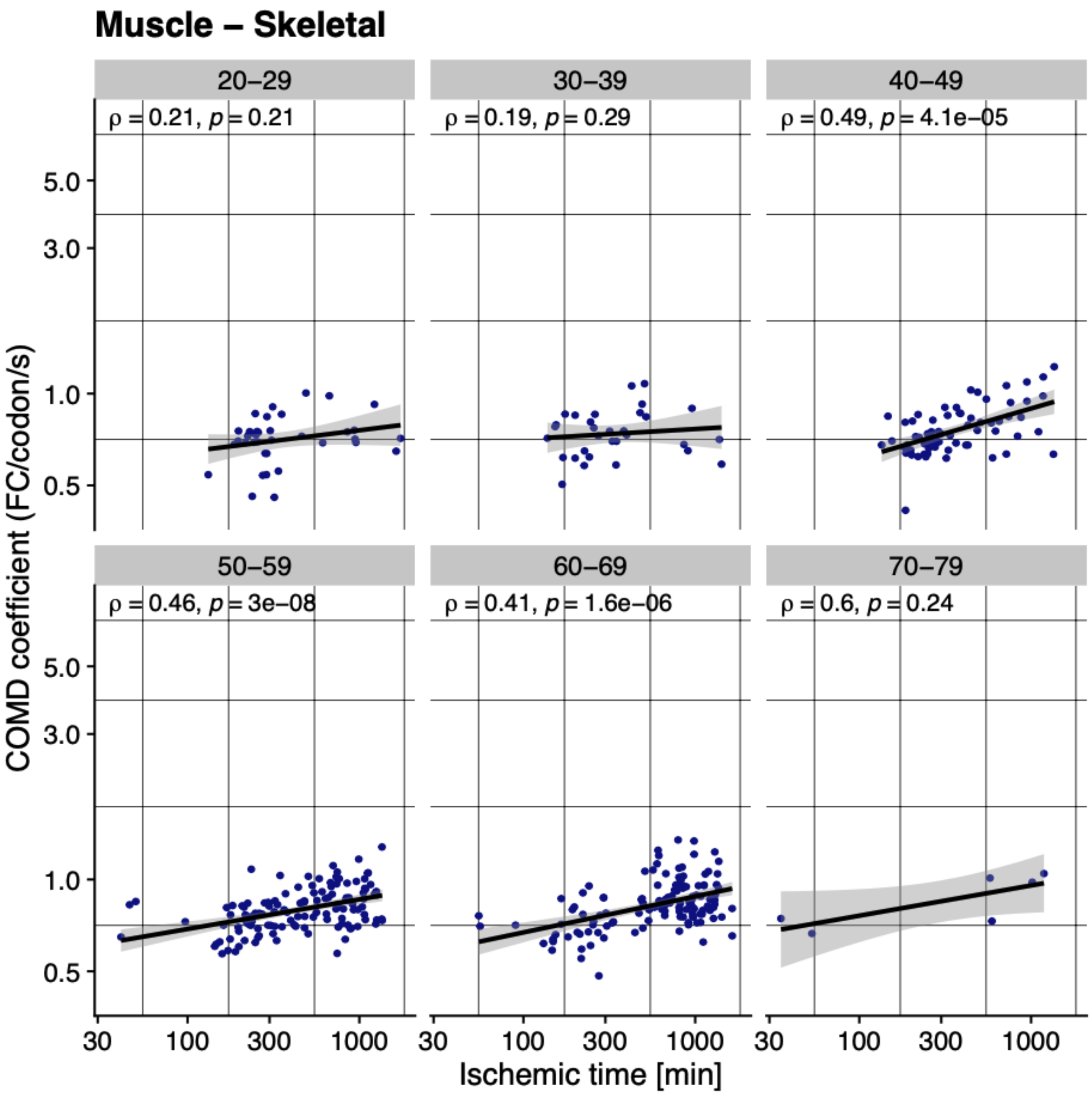

Appendix Figure S10: The COMD coefficient associates with ischemic time for individuals of similar age in Muscle - Skeletal.

COMD coefficient against ischemic time (min) for Muscle - Skeletal in different age groups.

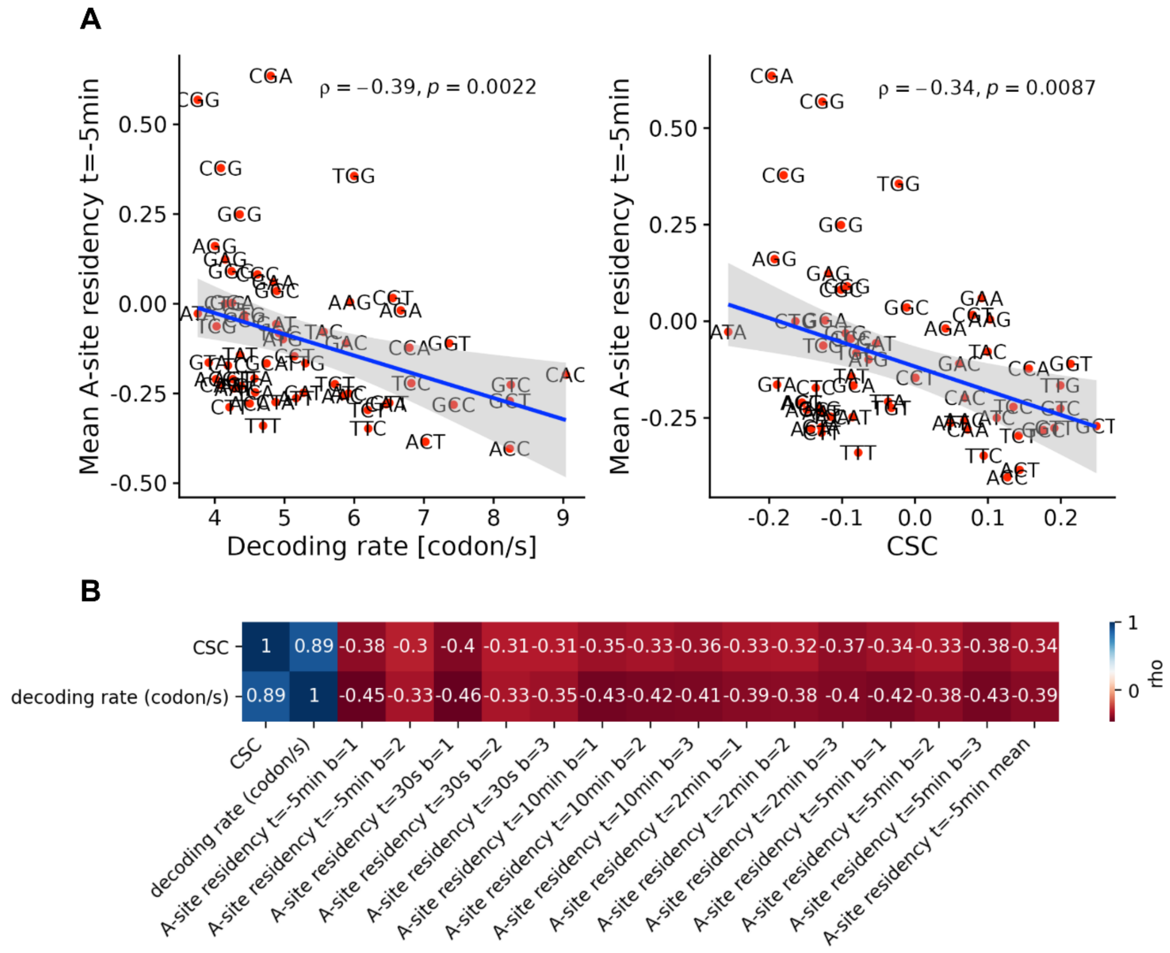

Appendix Figure S11: Codon-associated 5’ coverage correlates with codon optimality and decoding rate metrics.

A. Mean codon-associated 5’ coverage across batches at time point -5 min against CSC (codon stabilization coefficient, taken from (Presnyak et al, 2015)) and decoding rate (taken from (Dana & Tuller, 2015)).

B. Correlation between the codon-associated 5’ coverage of each sample (time point, *t*, and batch, *b*) and codon optimality or codon decoding rate.

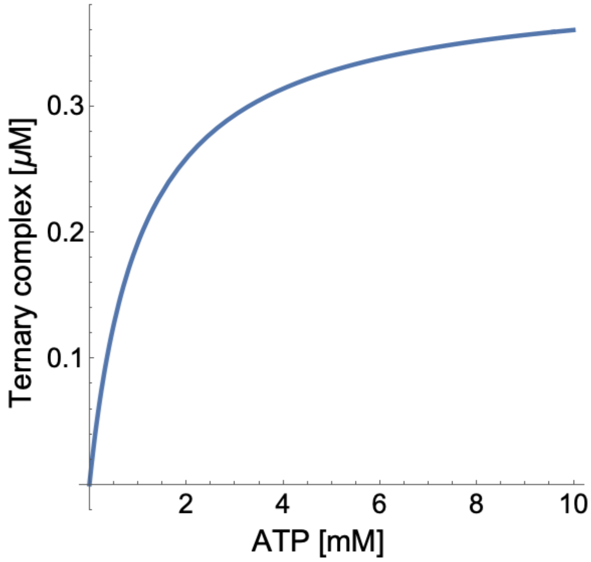

Appendix Figure S12: Ternary complex concentration increases asymptotically with ATP concentration.

Ternary complex against ATP concentration for the same rate constant values as in Figure 2F,G and tRNA and ribosome concentrations of 0.4μM and 0.1μM respectively.
